# Activation of the JNK-c-Jun pathway in response to irradiation facilitates Fas ligand secretion in hepatoma cells and increases hepatocyte injury

**DOI:** 10.1186/s13046-016-0394-z

**Published:** 2016-07-18

**Authors:** Yinying Dong, Xiaoyun Shen, Mingyan He, Zhifeng Wu, Qiongdan Zheng, Yaohui Wang, Yuhan Chen, Sifan Wu, Jiefeng Cui, Zhaochong Zeng

**Affiliations:** Department of Radiation Oncology, Zhongshan Hospital, Fudan University, 180 Feng Lin Road, Shanghai, 200032 People’s Republic of China; Liver Cancer Institute, Zhongshan Hospital, Fudan University & Key Laboratory of Carcinogenesis and Cancer Invasion, Ministry of Education, 180 Feng Lin Road, Shanghai, 200032 People’s Republic of China; Department of Radiology, Shanghai Cancer Center, Fudan University, Shanghai, 200032 People’s Republic of China

**Keywords:** Hepatocellular carcinoma, Irradiation, JNK-c-Jun, FasL, Paracrine, Hepatocyte injury

## Abstract

**Background:**

It is well established that some irradiated liver non-parenchymal cells secrete pro-inflammatory cytokines to facilitate the development of radiation-induced liver disease. However, little is known on whether the irradiated hepatoma cells-mediated non-irradiated hepatocyte injury occurs in HCC patients. Here, we elucidated the roles of the irradiated hepatoma cells in driving non-irradiated hepatocyte injury and its underlying mechanism.

**Methods:**

SMMC7721 cells were cultured and divided into irradiated (4-Gy X-ray, R) and non-irradiated (NR) groups. At 24th hour after irradiation, conditioned medium (CM) from these cultures was mixed with normal culture medium in specific proportions, and termed as 7721-R-CM and 7721-NR-CM. Following incubation with these CM compound, the biological characteristics of L02 cells related to liver cell injury including viability, apoptosis and liver dysfunction indices were comparatively analyzed. Simultaneously, the levels of proliferation- and apoptosis-related cytokines in irradiated and non-irradiated SMMC7721 cells were also measured. FasL as a cytokine with significantly differential expression, was selected to clarify its effects on L02 apoptosis. Subsequently, FasL expression following irradiation was examined in SMMC7721 and other HCC cells with varying malignant potentials, as well as in HCC tissues, the related mechanism of higher expression of FasL in irradiated HCC cells was further investigated.

**Results:**

Apoptosis and liver dysfunction indices were all significantly enhanced in L02 cells treated with 7721-R-CM, whereas viability was suppressed, compared to those with 7721-NR-CM stimulation. FasL was identified as a leading differential cytokine in the irradiated SMMC7721 cells. Higher proportion of apoptosis was also found in L02 cells following FasL incubation. A recombinant Fas-Fc protein, which blocks Fas-FasL interaction, ameliorated 7721-R-CM-induced apoptosis in L02 cells. FasL was highly expressed in a dose-dependent manner, and peaked at the 24th hour post-irradiation in different HCC cells and their culture supernatant. Meanwhile, phosphorylation levels of JNK, ERK, Akt, and p38 were all upregulated significantly in irradiated HCC cells. But, only JNK inhibition was validated to block radiation-induced FasL expression in HCC cells. c-Jun, the target transcription factor of JNK, was also activated.

**Conclusion:**

In HCC cells, the JNK-c-Jun pathway plays an important role in mediating irradiation- induced FasL expression, which may be critical in determining non-irradiated hepatocyte injury.

**Electronic supplementary material:**

The online version of this article (doi:10.1186/s13046-016-0394-z) contains supplementary material, which is available to authorized users.

## Background

Hepatocellular carcinoma (HCC) is a common cancer in China associated with poor prognosis. Following the initial observation that HCC is radiosensitive tumor and with subsequent developments in radiation technology, radiotherapy (RT) has become a feasible and promising treatment modality for each stage of the disease, and is currently one of the major methods used for palliative treatment of HCC. However, delayed liver damage, such as radiation-induced liver disease (RILD), characterized by early veno-occlusive disease (2 weeks to 4 months after RT), late onset of apoptosis and necrosis of hepatocytes, liver dysfunction, radiation fibrosis, is inevitable and sometimes lethal [1]. Therefore, finding a way to prevent both acute and late toxicity in liver injuries is of paramount importance.

The liver is a highly radiosensitive organ, and the threshold dose for whole-liver irradiation is reportedly between 20 and 30 Gy [2]. Given that hepatocytes are more radio-resistant than other cells [3], they are not the direct and initial target during radiation-induced liver injury. Although hepatic cell apoptosis resulting from radiation contributes to liver injury, research targeting on liver cells alone may not enable insight into all of the diverse pathological changes occurring in RILD. Which cell initiates the apoptotic signal, and what signaling pathways involve in RILD, are still largely unknown.

Growing evidences suggest that RILD involves a complex cascade of signal events between liver cells and the host stromal microenvironment. This crosstalk modulates or determines the occurrence and progression of RILD. Generally, the microenvironment of liver cells in HCC is composed of hepatoma cells, hepatic stellate cells, fibroblasts, invading inflammatory/immune cells, endothelial cells, and other non-cellular components such as growth factors, inflammatory cytokines etc. [4]. The important roles of stromal cells and inflammatory cytokines in RILD progression have been well documented in some literatures. Activated Kupffer cells produce and release numerous pro-inflammatory cytokines, which initiate the acute hepatic microvascular pathogenesis that leads to apoptosis of the hepatocytes [5]. Conditioned medium (CM) from irradiated Kupffer cells more strongly induces apoptosis of irradiated hepatocytes than normal medium because the CM contains higher levels of TNF-α [6]. Moreover, inflammation cytokines also participate in the development of RILD. TNF-α, IL-1, and IL-6, are mediators of the pathogenesis in the early phase of RILD [7]. Early veno-occlusive disease (VOD) after radiation is a distinguishing characteristic of RILD, and sinusoidal endothelial cell (SEC) injury has been traditionally postulated as the initiating lesion of VOD in RILD [8]. The number of apoptotic SECs increased significantly after 30 Gy of liver irradiation in a rat model, by contrast, apoptotic hepatocytes were only occasionally seen [9]. SEC injury results in microcirculatory blood flow disturbance and secondary injury to hepatocytes, causing liver dysfunction. Thereby, early activation of stroma cells and release of inflammatory cytokines may contribute to radiation-induced liver injury. However, little studies are carried out on the roles of hepatoma cells in the occurrence of RILD following HCC radiotherapy. The stress as radiation, chemotherapy and heat shock enhance the expression and secretion of cytokines in HCC cells including interleukins (ILs), interferons (IFNs), TNFs, colony-stimulating factors, growth factors and chemokines [10–13]. Here, we hypothesized that the irradiated hepatoma cells may release specific cytokines and contribute to the occurrence of RILD.

In the present study, we found that the irradiated hepatoma cells promoted the apoptosis of normal hepatocytes through the secretion of pro-apoptotic cytokines, among which, a significant difference was seen in the expression of FasL. Furthermore, we elucidated the molecular mechanism of irradiation-induced FasL expression in hepatoma cells. From a perspective view of the interactions between the irradiated HCC cells and normal hepatic cells, this study provides novel insights into the pathogenesis of radiation-induced liver injury in HCC patients.

## Methods

### Hepatoma cell and liver cell

MHCC97H and HCCLM3 cells, which have high malignant potential, were established at the Liver Cancer Institute of Fudan University [14]. Huh7 cells with low malignant potential were obtained from the Chinese Academy of Sciences (Shanghai, China). Hep3B and SMMC7721 cells with low malignant potential and the normal liver cells L02 were purchased from the American Type Culture Collection (ATCC) (Manassas, VA, USA). These hepatoma cells and liver cells were routinely maintained.

### Chemical reagents and kits

Alanine transaminase (ALT), aspartate transaminase (AST), albumin (ALB), malondi- aldehyde (MDA), and superoxide dismutase (SOD) kits were from the Nanjing Jiancheng Bioengineering Institute (Nanjing, China). SB203580, PD98059, LY294002 and SP600125 were obtained from Sigma (St Louis, MO, USA). Recombinant human soluble FasL and Fas-Fc protein (which inhibits FasL activity) were purchased from Alexis Corp. (San Diego, CA, USA).

### Patients

Two independent cohorts with a total of 50 HCC patients were enrolled in this study as previously described [15]. In one cohort, after about 2 months of liver radiotherapy, 22 liver cancer patients underwent hepatectomy between January 2005 and May 2013. None of the patients received chemotherapy during the period of surrounding pre-hepatectomy liver radiotherapy. In another cohort, 28 tumor tissue samples were consecutively collected from patients undergoing curative resection between January and October of 2010. All hepatectomies were performed at the Liver Cancer Institute, Zhongshan Hospital, Fudan University. Approval for this study was obtained from the Zhongshan Hospital Research Ethics Committee.

### Radiation

Cells were irradiated as described previously [16]. A dose of 2–8 Gy X-ray irradiation was delivered to cells in a single fraction, using a linear accelerator (Oncor; Siemens, Munich, Germany). When hepatoma cells grew and reached approximately 75 % confluency in a T75 flask, the flask was placed on the couch, and a 1.5-cm-thick bolus was used to correct the distribution of radiation. Irradiation parameters were as follows: beam energy, 6-MV photons; dose-rate, 3 Gy/min; source-surface distance, 100 cm; size of the radiation field, 20 × 20 cm^2^; gantry, 180°. Dosimetry was measured with a cylindrical ionization chamber prior to irradiation.

### Collection of conditioned medium

Hepatoma cells were exposed to X-ray firstly, then cells were washed thoroughly and cultured in serum-free low-glucose Dulbecco’s modified Eagle medium (DMEM) for 24 h, and their culture supernatant was collected. The same medium was incubated for 24 h in a T75 flask with non-irradiated liver cancer cells to serve as the control. The collected culture medium was centrifuged at 4500 g for 30 min at 4 °C. The supernatant was stored at −80 °C for further use. The protein concentration of the CM was measured by a bicinchoninic acid protein assay (Pierce, Rockford, IL, USA).

### Cell viability assay

About 200 μl of L02 cells (3 × 10^3^cells) in DMEM containing 10 % FBS were seeded into a 96-well plate. At the indicated time points, 10 μl of CCK-8 solution (Dojindo, Japan) was added to the cells and incubated for 1 h. The number of viable cells in each well was tested by absorbance at 450 nm.

### Detection of apoptosis by FITC-labeled annexin V

Early apoptotic changes were detected by fluorescein isothiocyanate (FITC)-Annexin V staining. Propidium iodide (PI) was used to discriminate between apoptotic and necrotic cells among the annexin-V-positive cells. L02 cells (1 × 10^6^) were washed then resuspended in 100 μl binding buffer solution (Annexin-V-FITC Kit, Immunotech). Annexin V-FITC (5 μl) and PI (5 μl) were then added to the cell suspension for a 10-min incubation followed by fluorescence-activated cell sorting (FACS) analysis. FACS files were analyzed using FlowJo version 9.5.2 (Tree Star, Ashland, OR). Gating strategies for apoptotic cell are shown in Additional file 1: Figure S1.

**Fig. 1:**
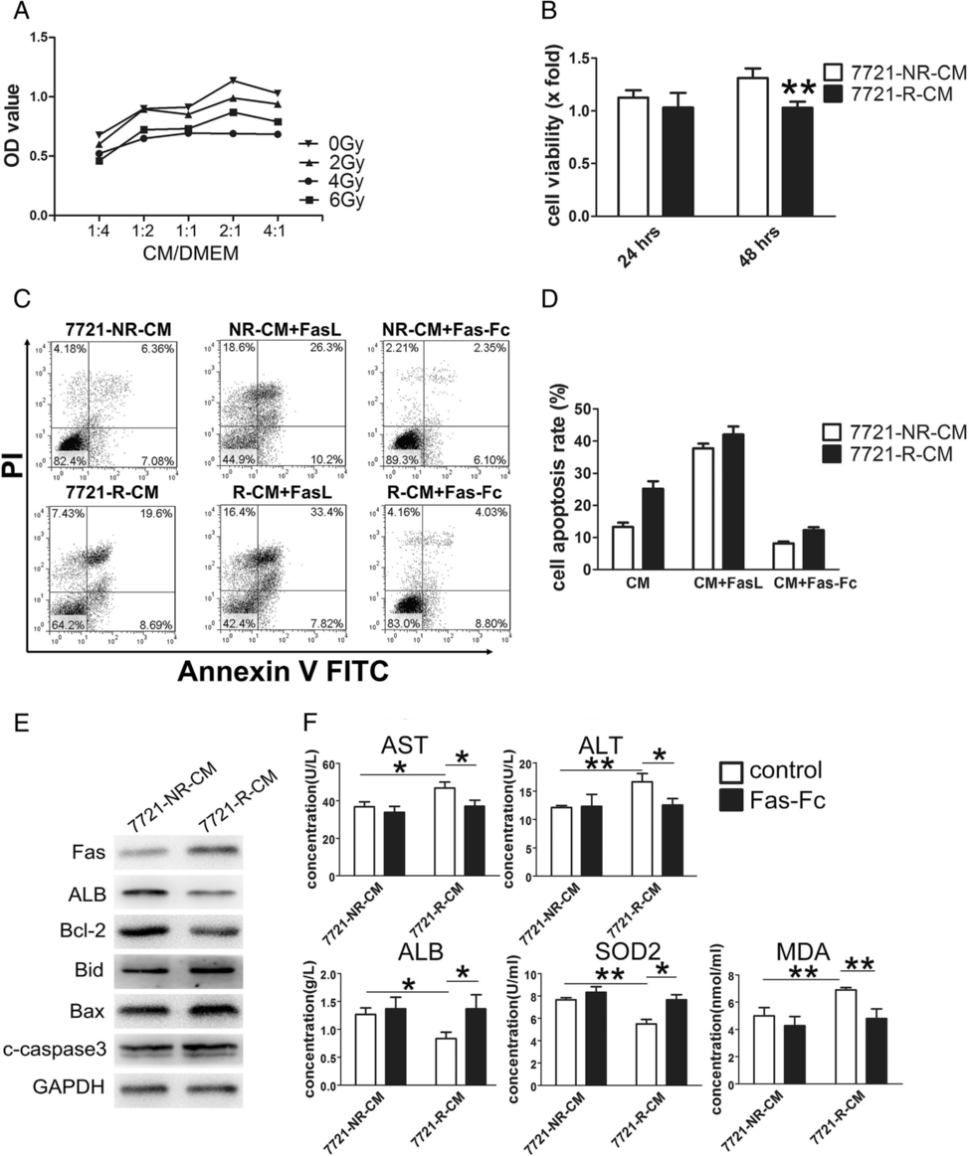
Conditioned medium derived from the irradiated HCC cells enhanced normal hepatocyte injury. **a** Conditioned media were collected from irradiated SMMC7721 cells (with exposure to 2, 4, or 6 Gray) and non-irradiated SMMC7721 cells after incubated for 24 h. Conditioned medium from each of the four groups was mixed with normal medium in different proportion (1:4, 1:2, 1:1, 2:1, and 4:1). Normal hepatocytes L02 were incubated with these mixtures for 48 h. The viability of L02 cells was examined by CCK8 assay. **b** Viability of L02 cells was examined by the CCK8 assay at different time points after incubation with 7721-NR-CM and 7721-R-CM. Data presented were fold-change of 7721-R-CM group compared with 7721-NR-CM group ± SD from three independent experiments. **P < 0.01. **c**, **d** L02 cells were cultured in different medium as indicated, and apoptotic cells were detected by flow cytometry after 24 h. **e** The protein levels of Fas, ALB, Bcl-2, Bid, Bax, and cleaved caspase3 were determined by Western Blot. GAPDH was used as a loading control. **f** Changes of AST, ALT, ALB, SOD2, and MDA levels in culture supernatant of L02 cells after incubated with 7721-R-CM and 7721-NR-CM alone or combined with Fas-Fc. *P < 0.05, **P < 0.01

### Quantitative reverse transcription polymerase chain reaction (qRT-PCR)

Total RNA extraction and qRT-PCR analysis were performed as our previous work [17]. Relative gene expression was normalized to glyceraldehyde phosphate dehydrogranase (GAPDH) and reported as 2^-ΔCt^ [ΔCt = Ct (FasL or other genes)-Ct (GAPDH)]. The primer sequences of all detected genes are listed in Table 1.

**Table 1:** Primer pairs used for qRT-PCR

Gene symbol	Sequence 5′-3′
FasL	Forward: 5′-TGCCTTGGTAGGATTGGGC- 3′
	Reverse: 5′-GCTGGTAGACTCTCGGAGTTC - 3′
TRAIL	Forward: 5′-TGCGTGCTGATCGTGATCTTC- 3′
	Reverse: 5′-GCTCGTTGGTAAAGTACACGTA- 3′
TNF-α	Forward: 5′-CCCGACTATCTCGACTTTGC- 3′
	Reverse: 5′-GGTTGAGGGTGTCTGAAGGA- 3′
IL-6	Forward: 5′-CAATGAGGAGACTTGCCTGG- 3′
	Reverse: 5′-GGCATTTGTGGTTGGGTCAG - 3′
IGF-1	Forward: 5′-GCTCTTCAGTTCGTGTGTGGA- 3′
	Reverse: 5′-GCCTCCTTAGATCACAGCTCC - 3′
PDGFA	Forward: 5′-GCAAGACCAGGACGGTCATTT- 3′
	Reverse: 5′-GGCACTTGACACTGCTCGT- 3′
PDGFB	Forward: 5′-CTCGATCCGCTCCTTTGATGA- 3′
	Reverse: 5′-CGTTGGTGCGGTCTATGAG - 3′
VEGFA	Forward: 5′-AGGGCAGAATCATCACGAAGT- 3′
	Reverse: 5′-AGGGTCTCGATTGGATGGCA - 3′
TGF-β1	Forward: 5′-AGGACTGCGGATCTCTGTGT - 3′
	Reverse: 5′-GGGCAAAGGAATAGTGCAGA - 3′
GAPDH	Forward: 5′- CTCCTCCACCTTTGACGC - 3′
	Reverse: 5′- CCACCACCCTGTTGCTGT- 3′

*qRT-PCR* quantitative real time reverse transcription polymerase chain reaction

### Western blot

Protein extraction and Western blot analysis were done as previously described [18]. Primary antibodies were diluted with 3 % TBSA as follows: ALB, Bcl-2, Bax, Bid, Fas, Akt, p-Akt(Ser473), p-ERK (Thr202/Tyr204), ERK, p-p38(Thr180/Tyr182), p38, caspase3, JNK, p-JNK(Thr183/Tyr185), c-JUN, p-c-JUN (Ser73), or GAPDH (1:1000, Cell Signal Technology, Danvers, MA), or FasL (1:500, Santa Cruz Biotechnology, Santa Cruz, CA, USA). Secondary antibodies were diluted with 3 % TBSA (against mouse and rabbit, 1:5000; Dingguo Bio, Beijing, China).

### Immunohistochemistry analysis

Immunohistochemical staining was performed based on the method of Wu [15]. In a typical procedure, after rehydration and antigen retrieval, cell slides were incubated with diluted primary antibodies against FasL (1:100, Santa Cruz) at 4 °C overnight, followed by HRP-conjugated secondary antibody (anti-rabbit, 1:200; DingguoBio) at 37 °C for 30 min. Finally, the slides were stained with 3,3′-diaminobenzidine (DAB) and counterstained with Mayer’s hematoxylin. Staining intensity and the percentage of immunoreactive tissues were scored by two independent observers, who were blinded to the patients’ outcomes. Five high-power fields (magnification, 200×) were randomly selected. Based on the IHC staining percentage and intensity of positive cells counted in each core, immunoreactivity was categorized as follows: negative (−), weak or mild (+), moderate (++), strong (+++), or stronger (++++), which corresponding successively to 0–4 points. The level of FasL expression in the two independent cohorts of HCC patients were compared.

### Immunofluorescence staining

Immunofluorescence staining was done as the method reported previously [17]. FasL (1:25, Santa Cruz, USA) antibody was diluted in 1 % bovine serum albumin (BSA). Secondary antibody was Alexa Fluor 488-conjugated goat anti-mouse antibody (Molecular Probes, Eugene, OR).

### Enzyme-linked immunosorbent assay (ELISA)

The level of FasL in cell culture supernatants was determined using the Quantikine Human FasL ELISA Kit (Abcam Systems) according to the manufacturer’s instructions. Briefly, 100 μL sample was added to each well and incubated for 2.5 h at room temperature. The plates were washed and incubated with the FasL conjugate for 2 h. After washing, immunoreactivity was determined by adding substrate solution and the absorbance was determined using a Microplate Spectrophotometer (Bio-Rad, Hercules, CA, USA). A curve of absorbance versus the concentration of FasL in the standard wells was plotted.

### Recombinant plasmid construction and transfection

To generate plasmid-expressing c-Jun-shRNA, double-stranded oligonucleotides were cloned into GV248 vector. The sequences of c-Jun-shRNA used are CcggcgGACCTTATGGCTACAGTAActcgag TTACTGTAGCCATAAGGTCCGTTTTTg.

The uppercase letters represent c-Jun-specific sequence, and lowercase letters represent hairpin sequences. SMMC7721 and MHCC97H were transfected with plasmid using lipofectamine 2000.

### Statistical analysis

Data were analyzed using SPSS software (version 16.0). Results were expressed as mean ± SD. Statistical analysis was performed by one-way ANOVA and Student’s t -test. *P* < 0.05 was considered statistically significant.

## Results

### CM derived from the irradiated HCC cells promoted normal hepatocyte injury

CM derived from the irradiated SMMC7721 cells with various dose irradiation (2, 4 and 6Gy) and CM from non-irradiated SMMC7721 cells were collected. Liver cells were treated with various concentrations of CM compound (CM mixed with completed DMEM in ratios of 1:4, 1:2, 1:1, 2:1, and 4:1) for 48 h. CCK8 assays showed that CM compound from the irradiated SMMC7721 cells reduced the viability of liver cells compared with that from non-irradiated cells. The most obvious difference occurred at the dose of 4Gy irradiation and media proportion of 2:1 (Fig. 1a). The CM compound from irradiated (4Gy) and non-irradiated SMMC7721 cells at ration of 2:1 with completed DMEM were termed as 7721-R-CM and 7721-NR-CM, respectively. We further evaluated their effects on the viability of liver cells. As shown in Fig. 1b, 7721-R-CM decreased cell viability in a time-dependent manner compared with 7721-NR-CM, and the liver cells treated with 7721-R-CM exhibited a reduction of about 20 % in viability (*P* < 0.01).

Cell apoptosis analysis showed that the proportion of early apoptosis and late apoptosis in the treated L02 cells (25.2 ± 5.82 %) with 7721-R-CM were remarkably increased as compared with that of the control with 7721-NR-CM (13.3 ± 2.53 %) (Fig. 1c, d). Simultaneously, expressions of pro-apoptotic protein Bax, Bid and cleaved caspase 3, which act as key executors in apoptosis and play important roles in programmed cell death, were upregulated in the treated L02 cells with 7721-R-CM, whereas the anti-apoptotic Bcl-2 was decreased (Fig. 1e, Additional file 2: Figure S2). Unprocessed and uncropped western blot pictures were supplied in Additional file 3: Figure S3.

Levels of AST, ALT, ALB, SOD2, and MDA in culture medium reflects the extent of live cell injury. As shown in Fig. 1f, 7721–R-CM-treated liver cells exhibited a significant elevation in the activities of ALT, AST and a depletion in ALB content. Meanwhile, the treated cell also had a significant decline in SOD2 levels and an increase in the concentration of MDA (Fig. 1f).

These data mentioned above suggest that culture medium from the irradiated HCC cells may contribute to the injury of liver cells by suppressing hepatocyte viability as well as inducing liver cell apoptosis and dysfunction.

### FasL in CM from the irradiated HCC cells may enhance liver cell injury

**Fig. 2:**
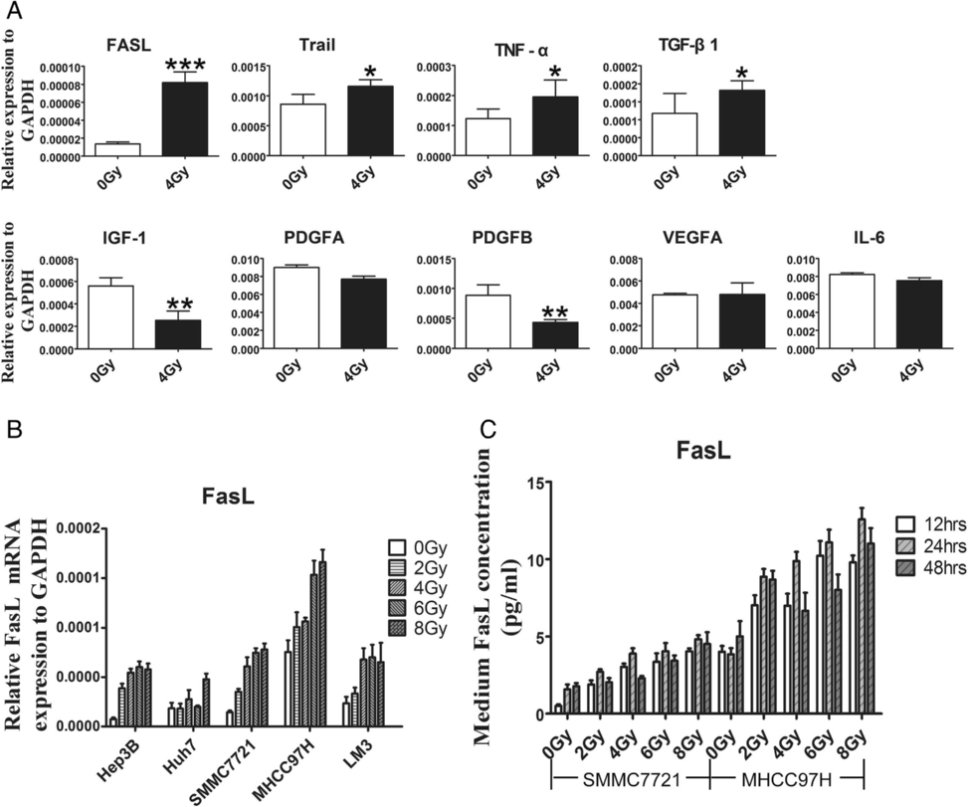
Irradiation altered the expressions of some proliferation and apoptosis- associated cytokines in SMMC7721 cells. **a** 24 h after irradiation, expressions of nine proliferation- and apoptosis- associated cytokines were examined using RT-PCR.*P < 0.05, **P < 0.01, ***P < 0.001. **b** Effect of radiation on FasL expression in human hepatoma cells with different malignant potentials was explored. Five types of hepatoma cells indicated were treated with single doses of 0-8Gy respectively, and incubated for 24 h. The expressions of FasL in five hepatoma cells were measured by RT-PCR. **c** SMMC7721 cells and MHCC97H cells, representing low and high malignant potential, as indicated, were treated with single doses of 0–8Gy, and incubated for 12, 24, and 48 h respectively, then the concentration of the secreted FasL in culture medium was measured by ELISA

The development of CM-induced liver cell injury in vitro indicates that irradiation might alter the production and secretion of certain factors that result in obvious changes in cell proliferation and apoptosis. Thereby, we comparatively analyzed gene expression levels of some known cytokines related to cell growth (transforming growth factor [TGF]-β1, vascular endothelial growth factor [VEGF] A, insulin-like growth factor [IGF]-1, IL-6, platelet-derived growth factor [PDGF] A, PDGFB) and death (FasL, TNF-related apoptosis-inducing ligand [TRAIL], TNF-α) between in irradiated SMMC7721 cells and in non-irradiated cells. Irradiation significantly upregulated the mRNA expressions of FasL, TRAIL, TNF-α, and TGF-β1 in hepatoma cells, and downregulated the mRNA expressions of IGF-1 and PDGFB (Fig. 2a), but no obvious effects on the expressions of VEGFA, PDGFA, and IL-6. Among these cytokines, FasL was identified as a leading differential expression cytokine in the irradiated HCC cells. We further speculated that FasL in 7721-R-CM may play an important role in the induction of liver cell injury. To confirm this notion, we pre-incubated L02 cells with 7721-R-CM and 7721-NR-CM for 18 h, and subsequently treated them with 50 ng/ml FasL for 6 h. As expected, FasL caused a significant increase in L02 cell death and caspase3 activation, as compared to treatment with only 7721-R-CM or 7721-NR-CM for 24 h. Moreover, we employed a recombinant Fas-Fc protein to block the interaction between Fas and FasL, and found that the inclusion of 1 μg /mL Fas-Fc in the medium significant reduced the rate of liver cell death and the expression of cleaved caspase3 compared with the control (Fig. 1c, d, Additional file 2: Figure S2). Simultaneously, Fas-Fc prevented 7721-R-CM-induced changes of AST, ALT, ALB, SOD2 and MDA (Fig. 1f). In addition, 7721-R-CM incubation upregulated Fas (FasL receptor) expression in L02 cells (Fig. 1e). These data suggest that FasL, secreted from the irradiated hepatoma cells promotes the injury of liver cells.

Subsequently, five types of hepatoma cells with different malignant potentials were used to validate changes in FasL levels following radiation. Hepatoma cells received a single dose of 0, 2, 4, 6, and 8 Gy radiation respectively and further incubated for 24 h. Higher radiation dose resulted in higher gene expression of FasL (Fig. 2b). In addition, the up-regulation of FasL in the irradiated hepatoma cells was independent of malignant potential, and the sensitivity to radiation was also different among different types of hepatoma cells.

**Fig. 3:**
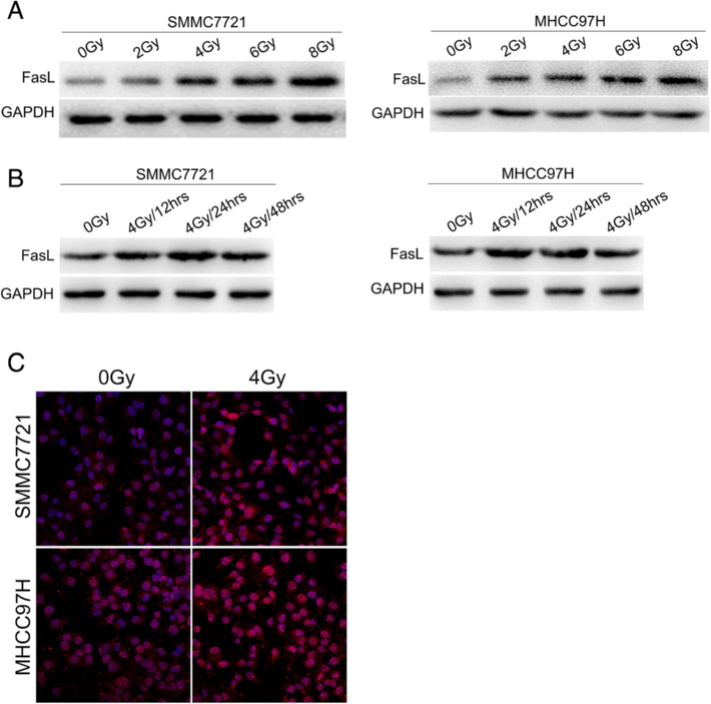
Irradiation upregulated FasL expression in SMMC7721 cells and MHCC97H cells. **a** Protein levels of FasL in HCC cells were determined after be irradiated with doses of 0, 2, 4, 6, 8Gy and incubated for 24 h. **b** FasL expression was detected in HCC cells after be irradiated with 4 Gy and incubated for 12, 24, and 48 h, respectively, by Western Blot. GAPDH was used as a loading control. **c** Increased expression of FasL was confirmed by immunofluorescent staining inHCC cells after be irradiated with 4Gy and incubated for 24 h

**Fig. 4:**
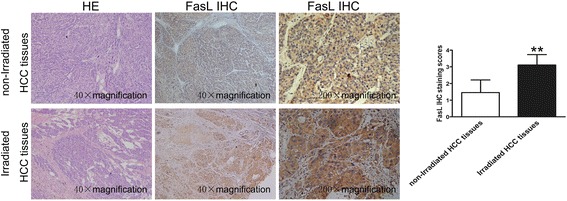
Effect of radiotherapy on FasL expression in HCC tissues. Representative images of FasL staining in HCC tissues with radiotherapy and without radiotherapy. FasL expression was analyzed by immunohistochemistry. Five randomly selected areas were viewed by light microscopy (200× total magnification). Data on the staining scores are shown as means ± SD. * *P < 0.01

MHCC97H cells and SMMC7721 cells with higher and lower malignant potential were further explored what occurred in the cell culture medium following irradiation. Be consistent with the results of qRT-PCR, FasL protein level also exhibited a dose- dependent manner with irradiation dose at 0, 2, 4, 6, and 8 Gy, levels of FasL were detectably higher at 12th hour after irradiation and reached a maximum at 24th hour, but no significant variations were observed between 12 and 48 h (Fig. 2c). These data were confirmed by western blot, in which a 39-kDa band corresponding to FasL protein was detectable in extracts from non-irradiated and irradiated MHCC97H and SMMC7721 cells (Fig. 3a,b). Unprocessed and uncropped western blot pictures were provided in Additional file 4: Figure S4. Confocal microscopy also supported that FasL expression in the cell cytoplasm was induced by irradiation (Fig. 3c). Two sets of HCC tissue samples were used to define the relationship between radiation and FasL expression. FasL expression in the radiation group was remarkably higher than that in the non-radiation group (Fig. 4), indicating a positive correlation between FasL expression and radiation.

Together, these data imply that radiotherapy may modulate the expression of FasL in HCC cells, further influence liver cell apoptosis.

### Radiation elevates the level of FasL in HCC cells via activating the JNK-c-Jun pathway

**Fig. 5:**
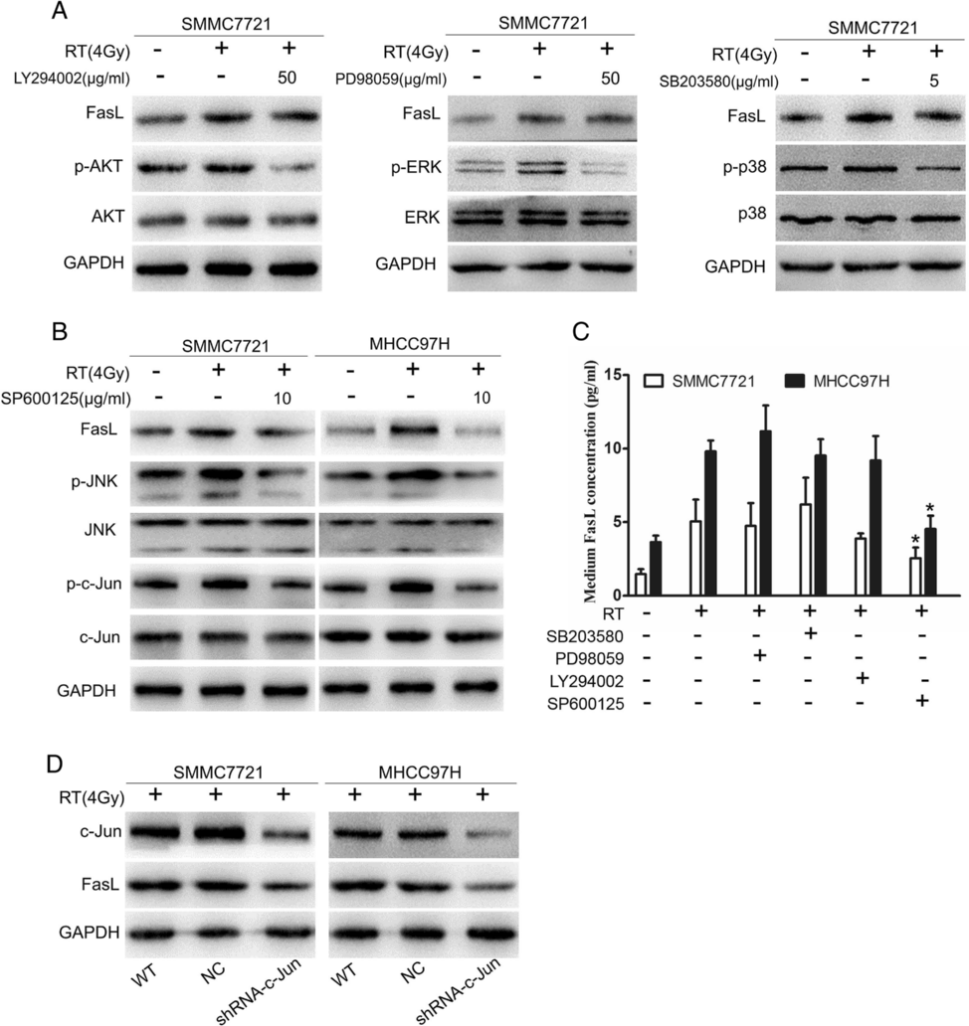
JNK-c-Jun signal pathway may contributes to radiation-enhanced FasL expression. **a** SMMC7721 cells were treated with chemical inhibitors, SB203580 (p38), PD98059 (MAPK/ERK), LY294002 (PI3K/Akt), and SP600125 (SAPK/JNK) respectively for 60 min before irradiation. FasL and phosphorylated forms of p38, Akt, ERK, JNK in HCC cells were analyzed after be irradiated with 4Gy exposure and incubated for 24 h. **b** Activation of the JNK-c-Jun signaling pathway was investigated by Western Blot. **c** The protein levels of FasL in culture supernatant were determined using ELISA. **d** FasL expression after c-Jun silencing. *P < 0.05

Radiation exposure is a kind of stress and may activate several intracellular signaling pathways, such as mitogen-activated protein kinase (MAPK) and phosphoinositol-3- kinase (PI3K) [19]. Here, we investigated the effects of radiation on activation of downstream molecules of MAPK and PI3K pathways including p38, extracellular signal-regulated kinase (ERK), c-Jun NH2-terminal kinase (JNK) and Akt. A dose of 4Gy radiation could promote p38, ERK, JNK, and Akt phosphorylation in SMMC7721 cells, but no effects on expression of p38, ERK, JNK, and Akt (Fig. 5a), indicating that all of signal molecules were activated.

We respectively used SB203580, PD98059, LY294002, and SP600125 to inhibit phosphorylation of p38, ERK, Akt, and JNK, and found that only JNK-specific inhibitor SP600125 suppress radiation-induced FasL expression in SMMC7721 cells (Fig. 5a, b). This finding suggests that radiation may activate MAPK and its downstream kinase JNK, and contribute to radiation-induced FasL expression.

The c-Jun transcription factor is an important and specific target for JNK [20], therefore, we further analyzed the effect of radiation (4Gy) on the activation of c-Jun. Indeed, we observed phosphorylation of c-Jun, which could be blocked by the inhibitor of JNK. Consistent with the observations in SMMC7721 cells, activation of JNK-c-Jun signaling pathway also participated in radiation-induced FasL expression in MHCC97H cells (Fig. 5b). Additionally, the alteration of FasL levels in culture medium was similar to the changes in HCC cells (Fig. 5c). In order to confirm the effect of c-Jun on FasL expression, we transfected shRNA-c-Jun intoSMMC7721 and MHCC97H cells. As shown in Fig. 5d, FasL expression was decreased significantly in hepatoma cells with lower c-Jun expression. Unprocessed western blot pictures of Fig. 5a, b, d were supplied in Additional files 5 and 6: Figures S5 and S6. All above data suggest that radiation upregulates FasL expression in hepatoma cells through activating the JNK-c-Jun signaling pathway.

## Discussion

Results of the present study indicate that irradiated HCC cells and their surrounding non-irradiated liver cells could exhibit crosstalks, which results in a significant increase in hepatocyte injury. Liver cell injury was mainly evaluated by means of hepatocyte apoptosis and liver cell function.

Apoptosis, as one of the terminal pathways of the cell cycle, is a typical form of programmed cell death. It occurs in morphogenesis during embryonic development and in the elimination of aged or harmful cells for adult tissue homeostasis maintenance [21]. Apoptosis of hepatic cells is considered to be a prominent pathological feature in most forms of liver injury such as chronic viral hepatitis, cholestatic liver disease, and acetaminophen-induced hepatotoxicity [22], it is also a feature of irradiated hepatoma cell-induced non-irradiated liver cell injury. Cytokines, death ligands trigger caspase activation and induce cell apoptosis [23]. TNF-α and FasL-induced mouse hepatitis models exhibit activation of effector caspase-3 [24]. Bax, Bid and Bcl-2 are also associated with apoptosis pathways. Bax and Bid induce alterations in the mitochondrial permeability barrier by inserting into the mitochondrial membrane and driving the release of caspase-activating proteins from these organelles, like cytochrome c [25]. Bcl-2 can prevent induction of apoptosis caused by a wide variety of stimuli including cytotoxic cytokines, gamma irradiation, and chemotherapeutic drugs [26]. Bcl-2 controls a late event in a final common pathway during programmed and apoptotic cell death [27]. Several factors could reduce Bcl-2 levels and elevate Bax levels to induce cell apoptosis [28]. Our data demonstrated that 7721-R-CM promoted L02 apoptosis, upregulated the expressions of cleaved caspase-3, Bax and Bid, whereas suppressed the expression of Bcl-2, implying that some secreted cytokines in 7721-R-CM may drive or induce the occurrence of apoptosis of liver cells. On the other hand, 7721-R-CM also resulted in higher activities of AST, ALT and decrease of ALB expression in culture media of hepatocytes, which indicate a severe injury of liver cell function. Superoxide dismutase 2 (SOD2) is essential in detoxifying superoxides generated in the mitochondrial matrix and protecting mitochondria from reactive oxygen species (ROS), and is sensitive to liver injury [29]. As expected, 7721-R-CM downregulated the expression of SOD2 and increased the level of MDA in the supernatant of hepatocytes, suggesting that an increase of lipid peroxidation leading to cell damage, and the failure of antioxidant defense mechanisms [30]. Thereby, the treated liver cells with 7721-R-CM can better mirror the changes of liver injury, and there exists a positive correlation between the extent of caspase activation and the grade of liver injury.

In general, the death receptor-dependent (extrinsic) pathway and the mitochondrial-dependent (intrinsic) pathway are all involved in the regulation of cell apoptosis [22], and HCC cells also express diverse biological factors with pro- or anti-apoptotic activities [31]. Here, we also mentioned that the most obvious difference in liver cell viability occurred at the dose of 4Gy irradiation and media proportion of 2:1, but not at 6 Gy and 4:1 (Fig. 1a). This result indicates that radiation not only enhances pro-apoptotic factors level, but also upregulates proliferation factors like TGFβ1 (Fig. 2a). Other study also shows that radiation induces proliferation factor VEGF-C expression in lung cancer [32]. These secreted cytokines with different function influence cell proliferation or apoptosis together in conditioned medium from irradiated hepatoma cells. Taken together, the secreted cytokines from irradiated hepatoma cells may alter the microenvironment of the hepatocytes, and cause injury of the non-irradiated liver cells.

FasL was identified in this study as a leading differential cytokine between in the irradiated hepatoma cells and in non-irradiated cells. It is a transmembrane type II protein belonging to the TNF protein superfamily, which is also an important member of the death receptor-mediated extrinsic pathways [33]. Radiation increases FasL expression in many tumor types such as lymphoma, breast cancer, liver cancer and nasopharyngeal carcinoma [34–37]. So, we speculated that irradiated liver cancer cells could express FasL and mediate apoptosis of non-irradiated liver cells. Our data showed that FasL enhanced the apoptosis of L02 cells, and the Fas-Fc protein, which inhibits FasL-Fas interactions, ameliorated cell apoptosis, suggesting that FasL may play an important role in the apoptotic effect of irradiated hepatoma cells on normal hepatocytes. These results were consistent with those of other studies [38], supporting that FasL/Fas signaling pathway stimulates apoptosis in liver cells.

In this study, radiation increased FasL expression in a dose­dependent manner, and activated PI3K/Akt, MAPK/ERK, JNK, and p38, but only inhibition of JNK decreased the expression of FasL in hepatoma cells, indicating that radiation may upregulate the expression of FasL via JNK signaling pathway. The results were in agreement with other studies. Ectopic expression of MEKK1, regulated by the JNK pathway, leads to apoptosis and FasL expression in Jurkat T cells [20]. Upon cisplatin treatment, sustaining activation of JNK and p38 kinase (p38K) promotes upregulation of FasL in TR-4 cells [39]. JNK are key mediators of stress signals and seem to be responsible for protective responses, stress-dependent apoptosis, and inflammatory responses [40]. Various stresses such as UV and γ-irradiation, osmotic stress, and heatshock, as well as by pro-inflammatory cytokines (tumor necrosis factor-α, interleukin-1) and chemotherapeutic drugs can stimulate JNK signaling pathway [41]. Several transcription factors, such as c-Myc, nuclear factor (NF)-kB, and activating protein (AP)-1, are involved in regulation of FasL expression. UV irradiation upregulated FasL expression through NF-kB and AP-1 activation in Jurkat T cells [42]. Heterodimeric AP-1 complex consists of c-Jun and activating transcription factor 2 (ATF2) [43]. Similar with these reports, c-Jun activation in irradiated hepatoma cells also played important roles in enhancing FasL expression. Hereby, JNK-c-Jun signaling pathway may contribute higher expression of FasL in hepatoma cells exposed to X-ray irradiation.

## Conclusions

**Fig. 6:**
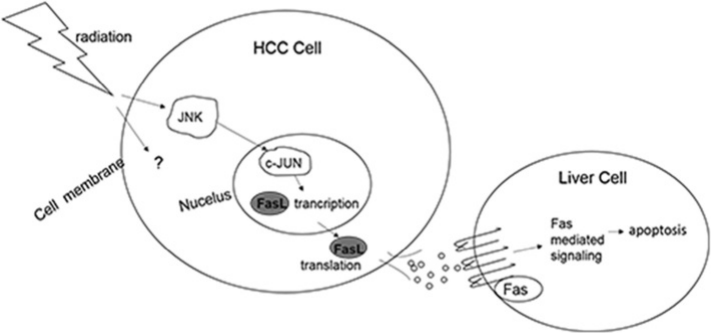
Schematic of mechanism. Diagram of the proposed mechanism by which irradiation initiates the JNK-c-Jun pathway to upregulate FasL expression and results in normal hepatocyte injury

In summary, this study constitutes the first observation that several secreted factors from irradiated HCC cells directly trigger apoptosis in liver cells. The data strongly suggest an involvement of JNK-c-Jun signaling pathway in irradiation-induced higher expression of FasL in hepatoma cells, and its roles in radiation-induced liver injury. Our study proposes a new paradigm (Fig. 6) that radiotherapy-induced FasL could be a paracrine apoptotic stimulus to accelerate radiation-induced liver injury.

## Abbreviations

ALB, albumin; ALT, alanine transaminase; AP-1, activating protein 1; AST aspartate transaminase; ATF, activating transcription factor; BSA, bovine serum albumin; CM, conditioned medium; ELISA, enzyme-linked immunosorbent assay; ERK, extracellular signal-related kinase; FACS, fluorescence-activated cell sorting; FITC, fluorescein isothiocyanate; GAPDH, glyceraldehyde phosphate dehydrogenase; HCC, hepatocellular carcinoma; IFN, interferon; IGF, insulin-like growth factor; IL, interleukin; JNK, c-Jun N-terminal kinase; MAPK, mitogen-activated protein kinase; MDA, malondialdehyde; NF-kB, nuclear factor kappa B; PBS, phosphate-buffered saline; PDGF, platelet-derived growth factor; PI, propidium iodide; PI3K, phosphoinositol-3-kinase; qRT-PCR, quantitative reverse transcription polymerase chain reaction; RILD, radiation-induced liver disease; ROS, reactive oxygen species; SEC, sinusoidal endothelial cell; SOD, superoxide dismutase; TBSA, the mixture formed by dissolving BSA with TBST to the required concentration; TGF, transforming growth factor; TNF-α, tumor necrosis factor; TRAIL, TNF-related apoptosis-inducing ligand; VEGF, vascular endothelial growth factor; VOD, veno-occlusive disease.

## Additional files

Additional file 1: Figure S1. FACS gating strategy of Annexin V/PI staining. (a) AnnexinV/PI staining was presented for population after gating by forward/side scattering. (b) Negative control, which is unstained sample to decide cutoff line for negative cells. The combination of Annexin V-FITC and propidium iodide (PI) allows for the distinction between early apoptotic cells (Annexin V-FITC positive), late apoptotic and/or necrotic cells (Annexin V-FITC and PI positive), and viable cells (unstained). (c) Liver cells were detected according to the cutoff line for negative cells to identify early apoptotic cells, late apoptotic and/or necrotic cells and viable cells. (JPG 349 kb) Additional file 2: Figure S2. Changes of caspase-3 activation in L02 cells after different treatment. L02 cells were cultured in different medium as indicated, and cleaved caspase3 were detected by flow cytometry after 24 h. (a) Data shown are representative histograms from each group of cells for three separate experiments. (b) The percentage of positive cells was indicated. (JPG 149 kb) Additional file 3: Figure S3. Uncropped and unprocessed immunoblot pictures of Fig. 1E. (JPG 101 kb) Additional file 4: Figure S4. Uncropped and unprocessed immunoblot pictures of Fig. 3A, B. (JPG 556 kb) Additional file 5: Figure S5. Uncropped and unprocessed immunoblot pictures of Fig. 5A, D. (JPG 1202 kb) Additional file 6: Figure S6. Uncropped and unprocessed immunoblot pictures of Fig. 5B. (JPG 613 kb)
