# Supplementary figures and images for: Activation of the JNK-c-Jun pathway in response to irradiation facilitates Fas ligand secretion in hepatoma cells and increases hepatocyte injury
Source: J Exp Clin Cancer Res. 2016 Jul 18;35:114. doi: 10.1186/s13046-016-0394-z (PMC4950705; doi:10.1186/s13046-016-0394-z)

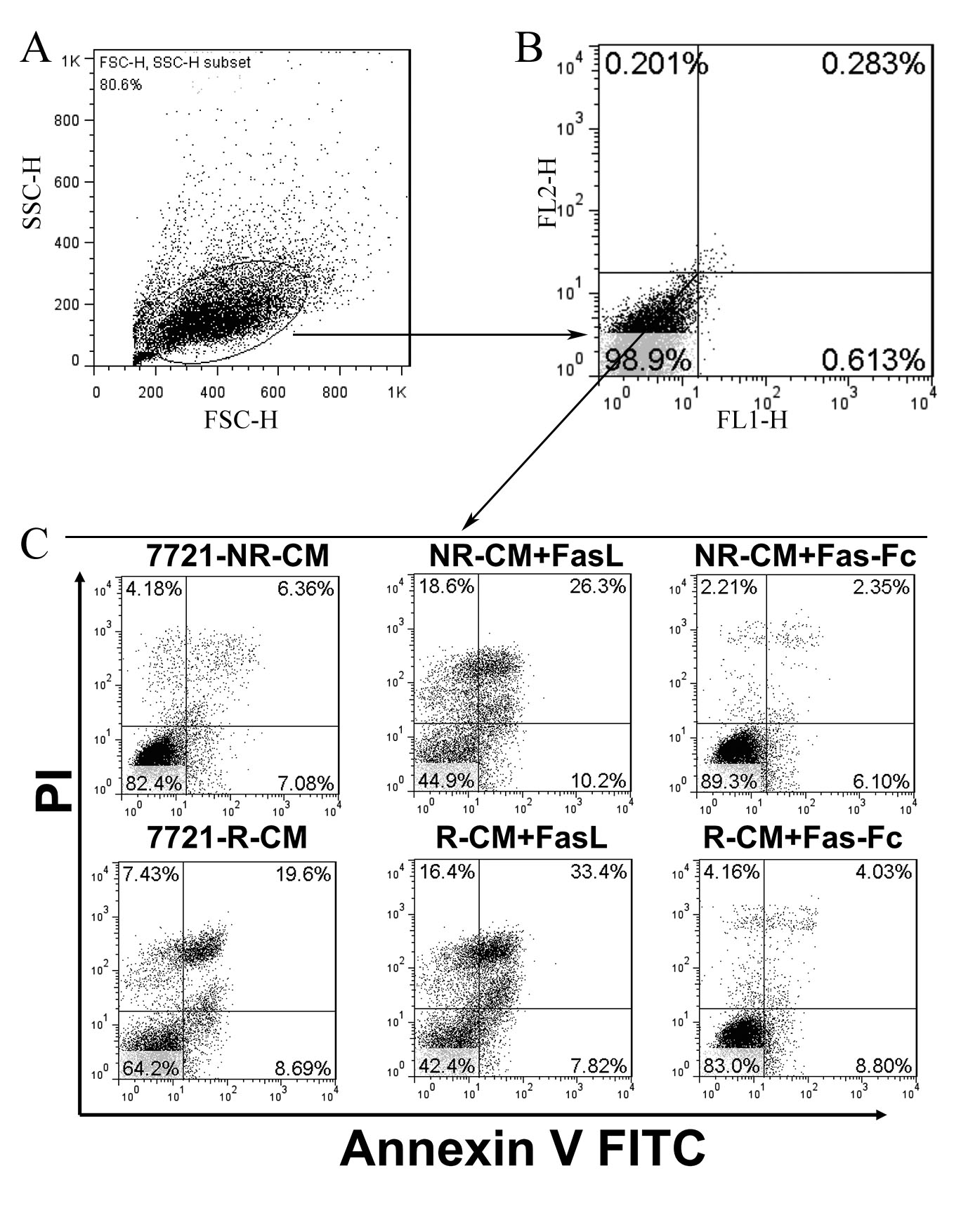

Supplement: Additional file 1: Figure S1. — FACS gating strategy of Annexin V/PI staining. (a) AnnexinV/PI staining was presented for population after gating by forward/side scattering. (b) Negative control, which is unstained sample to decide cutoff line for negative cells. The combination of Annexin V-FITC and propidium iodide (PI) allows for the distinction between early apoptotic cells (Annexin V-FITC positive), late apoptotic and/or necrotic cells (Annexin V-FITC and PI positive), and viable cells (unstained). (c) Liver cells were detected according to the cutoff line for negative cells to identify early apoptotic cells, late apoptotic and/or necrotic cells and viable cells. (JPG 349 kb) [file 13046_2016_394_MOESM1_ESM.jpg]

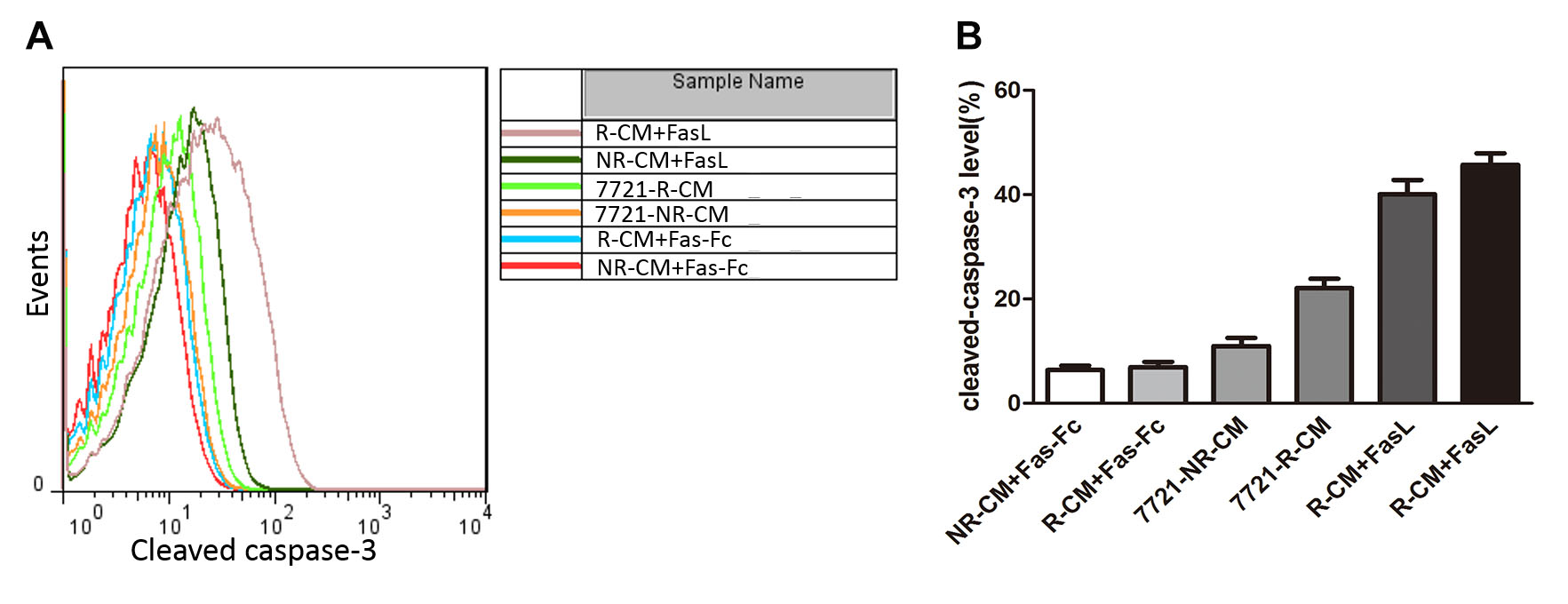

Supplement: Additional file 2: Figure S2. — Changes of caspase-3 activation in L02 cells after different treatment. L02 cells were cultured in different medium as indicated, and cleaved caspase3 were detected by flow cytometry after 24 h. (a) Data shown are representative histograms from each group of cells for three separate experiments. (b) The percentage of positive cells was indicated. (JPG 149 kb) [file 13046_2016_394_MOESM2_ESM.jpg]

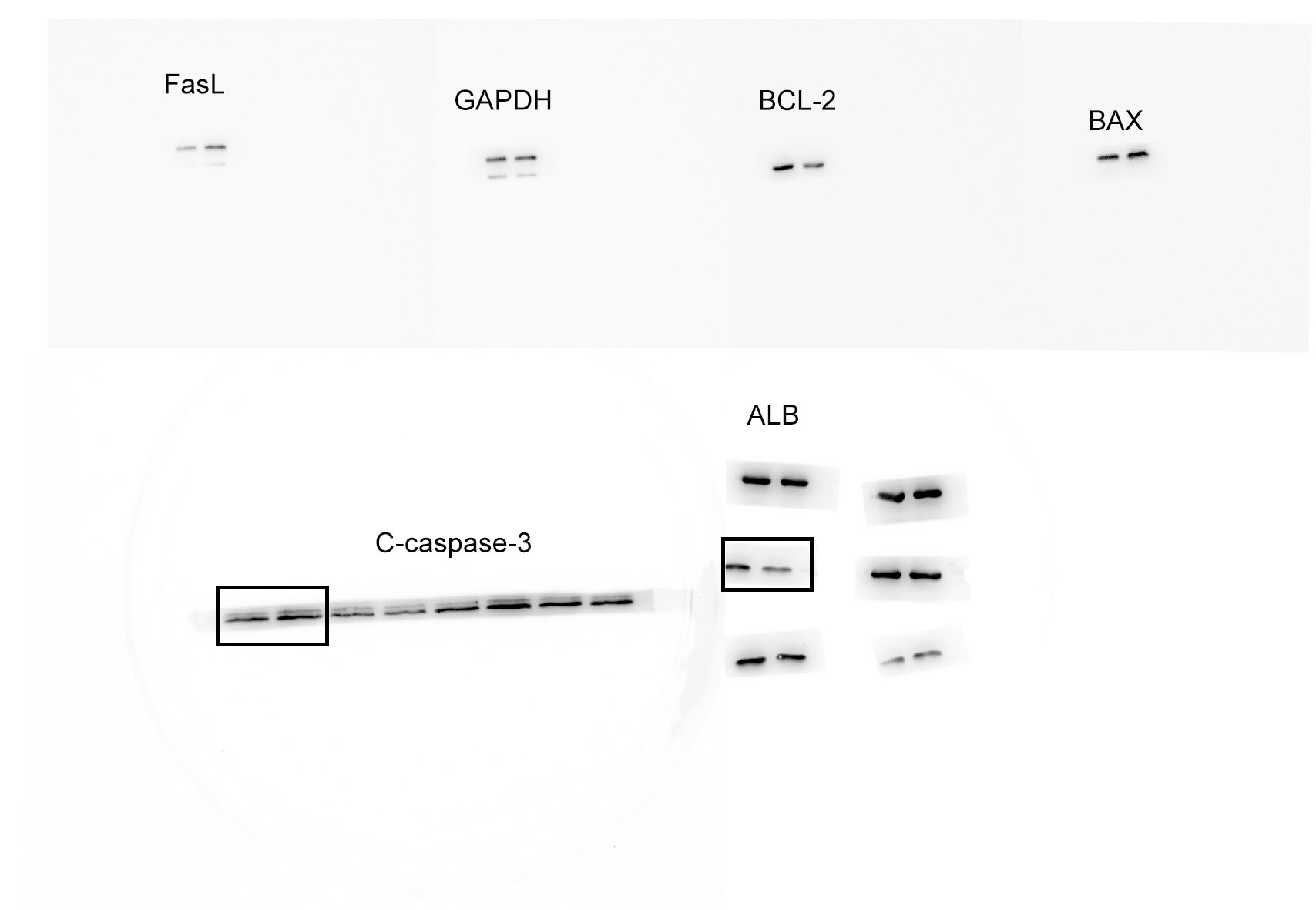

Supplement: Additional file 3: Figure S3. — Uncropped and unprocessed immunoblot pictures of Fig. 1E. (JPG 101 kb) [file 13046_2016_394_MOESM3_ESM.jpg]

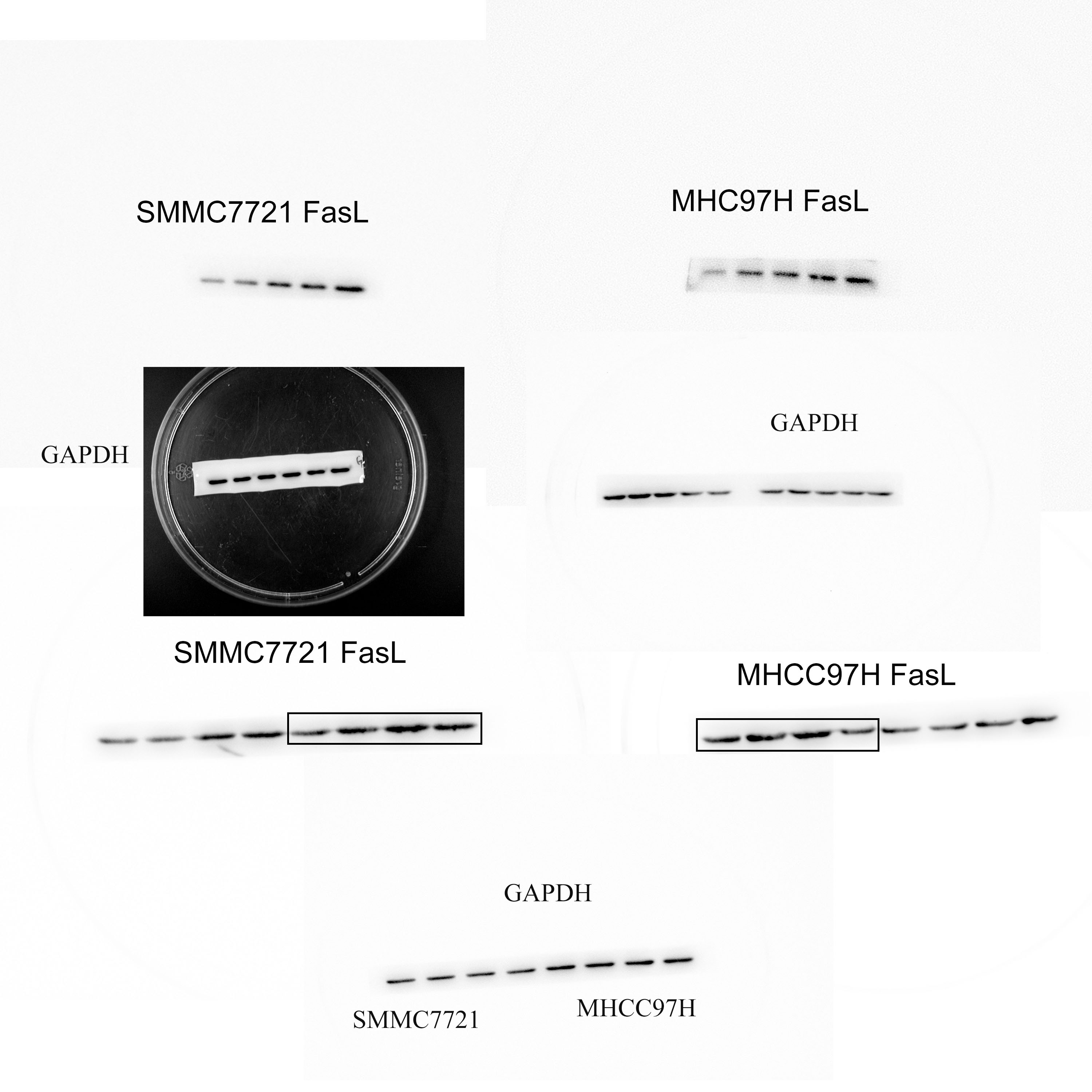

Supplement: Additional file 4: Figure S4. — Uncropped and unprocessed immunoblot pictures of Fig. 3A, B. (JPG 556 kb) [file 13046_2016_394_MOESM4_ESM.jpg]

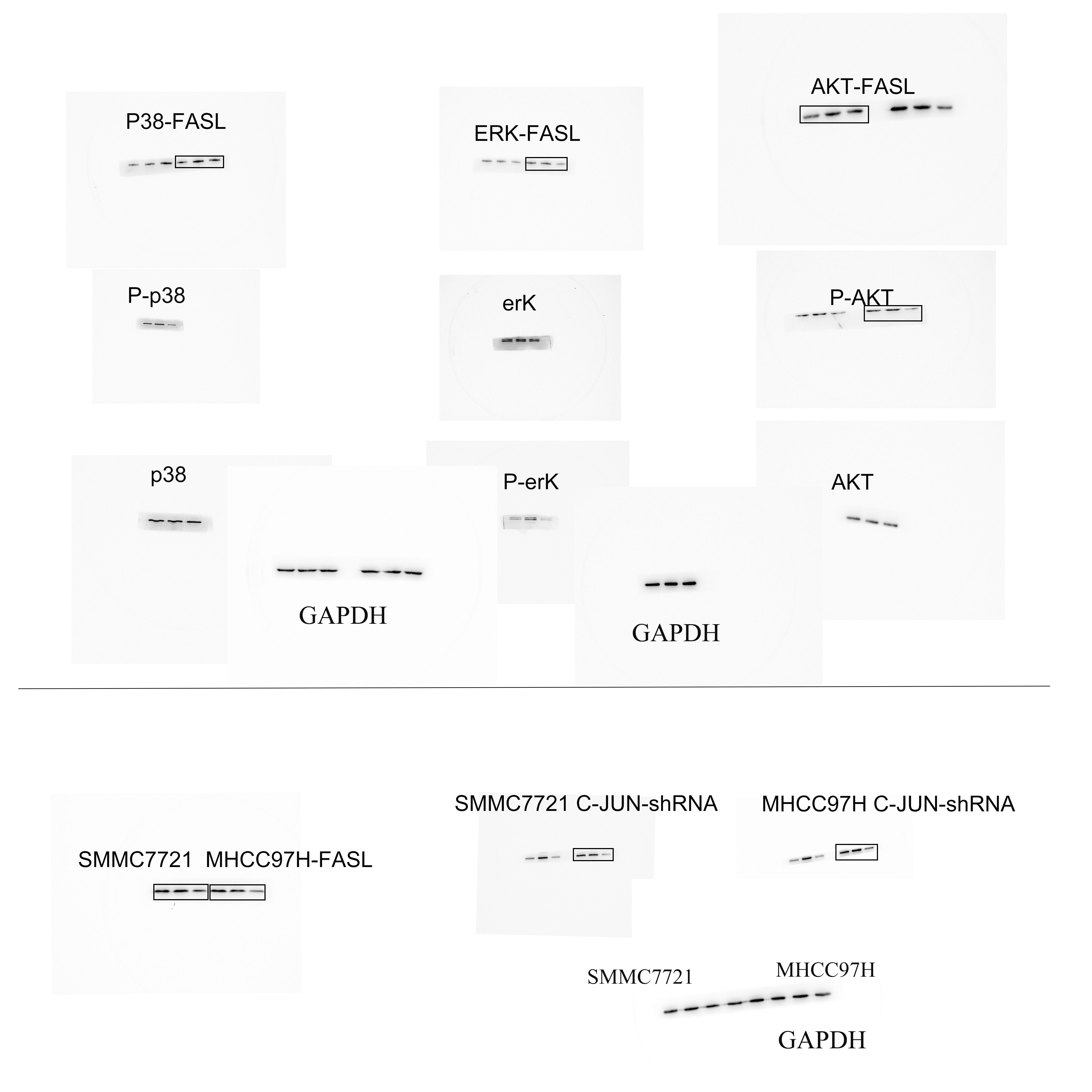

Supplement: Additional file 5: Figure S5. — Uncropped and unprocessed immunoblot pictures of Fig. 5A, D. (JPG 1202 kb) [file 13046_2016_394_MOESM5_ESM.jpg]

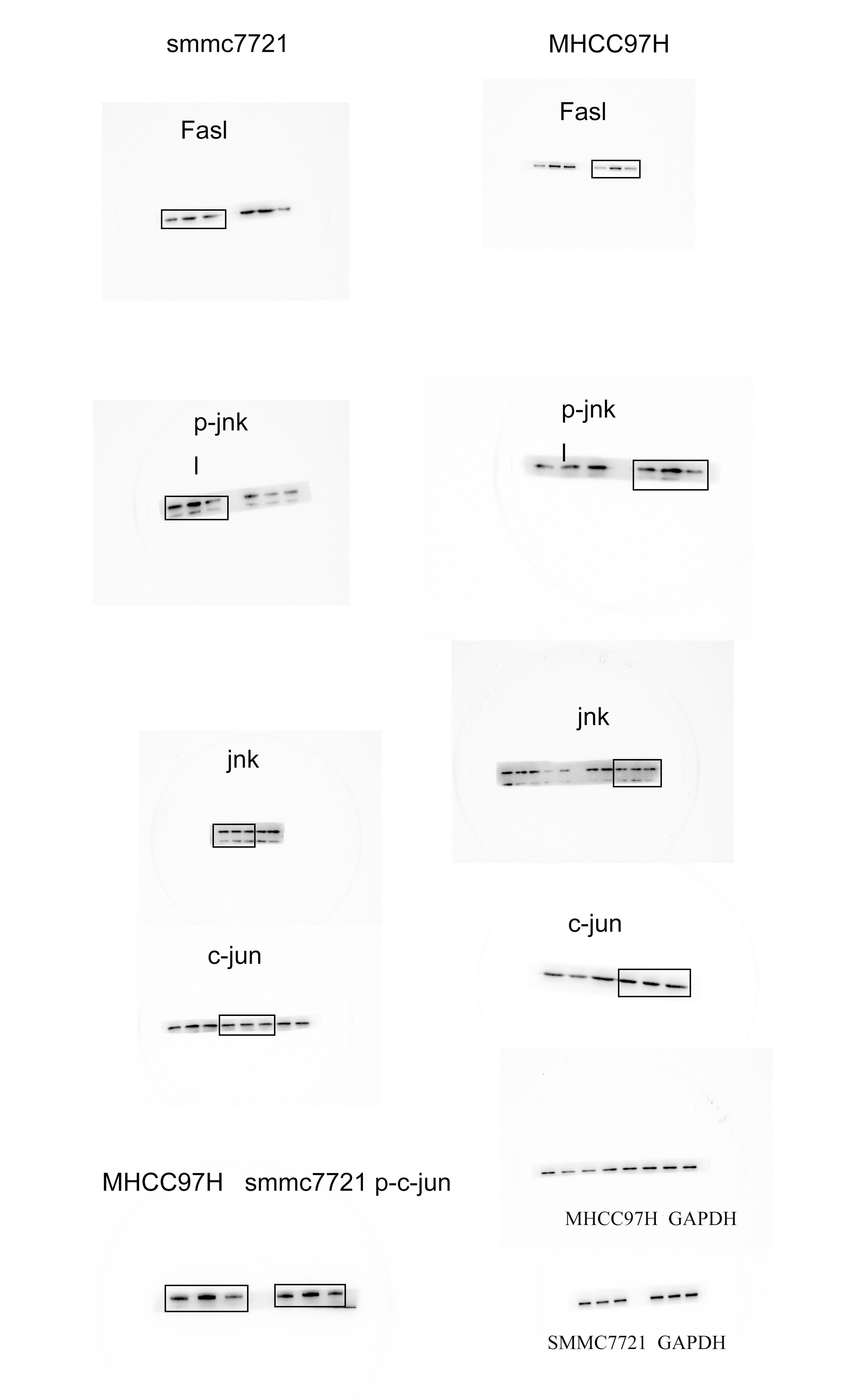

Supplement: Additional file 6: Figure S6. — Uncropped and unprocessed immunoblot pictures of Fig. 5B. (JPG 613 kb) [file 13046_2016_394_MOESM6_ESM.jpg]
